# Discovery of Chitin Deacetylase Inhibitors through Structure-Based Virtual Screening and Biological Assays

**DOI:** 10.4014/jmb.2201.01009

**Published:** 2022-02-05

**Authors:** Yaodong Liu, Sibtain Ahmed, Yaowei Fang, Meng Chen, Jia An, Guang Yang, Xiaoyue Hou, Jing Lu, Qinwen Ye, Rongjun Zhu, Qitong Liu, Shu Liu

**Affiliations:** 1 Jiangsu Key Laboratory of Marine Bioresources and Environment, Jiangsu Ocean University, Lianyungang, 222005, P.R. China; 2 Co-Innovation Center of Jiangsu Marine Bio-industry Technology, Jiangsu Ocean University, Lianyungang 222005, P.R. China; 3 University of California San Diego, 9500 Gilman Drive, La Jolla, CA 92093, USA; 4 Jiangsu Marine Resources Development Research Institute, Jiangsu Ocean University, Lianyungang 222000, P.R. China; 5 Lianyungang Inspection and Testing Center for Food and Drug Control, P.R. China

**Keywords:** Chitin deacetylase, enzyme inhibitor, virtual screening, biological assays

## Abstract

Chitin deacetylase (CDA) inhibitors were developed as novel antifungal agents because CDA participates in critical fungal physiological and metabolic processes and increases virulence in soilborne fungal pathogens. However, few CDA inhibitors have been reported. In this study, 150 candidate CDA inhibitors were selected from the commercial Chemdiv compound library through structure-based virtual screening. The top-ranked 25 compounds were further evaluated for biological activity. The compound J075-4187 had an IC50 of 4.24 ± 0.16 μM for *An*CDA. Molecular docking calculations predicted that compound J075-4187 binds to the amino acid residues, including active sites (H101, D48). Furthermore, compound J075-4187 inhibited food spoilage fungi and plant pathogenic fungi, with minimum inhibitory concentration (MIC) at 260 μg/ml and minimum fungicidal concentration (MFC) at 520 μg/ml. Therefore, compound J075-4187 is a good candidate for use in developing antifungal agents for fungi control.

## Introduction

Fungi are one of the largest groups of organisms, with almost 1.5 million species [1]. They constitute a prolific resource for bioactive metabolites used as beneficial antibiotics and drugs [2]. However, some fungi cause food spoilage, plant diseases, and people or animal diseases, which often result in heavy loss of life and property [3, 4].

The mycotoxins produced by fungi cause significant harm to livestock, domestic animals, and humans [5]. Furthermore, plant pathogenic fungi have strong adaptability and can enter a dormant state when the external environment is not suitable for growth, resulting in crop loss or even no harvest [6-8]. Still, each year, around 600 million people are fed by controlling the fungi that are also capable of destroying crops [9].

The invention of fungicides or antifungals has made it possible for humans to effectively control fungal threats. It has also led to an antifungals/fungicide industry worth around $30 billion globally [10]. There are tight regulations around the use of fungicides and antifungals, and there is also a growing resistance of fungi to these agents [11]. Resistance to fungicides and antifungals is a significant challenge for researchers worldwide [12]. Due to the lack of new fungicides or antifungal development, a growing number of researchers are working on new, safe therapeutic compounds. Enzyme inhibitor targets have demonstrated promise as future new antifungal drugs [13-15]. Moreover, new antifungal discoveries must be encouraged to avoid a global collapse in our ability to control harmful fungi.

Structure-based virtual screening (SBVS) has been developed as an effective method for identifying novel hits in drug discovery [16]. Compared with the traditional screening techniques, SBVS technology offers faster computing speed and lower cost than the experimental chemical screening of large databases. Based on the precise knowledge of the available crystal structures of enzymes, some potent new enzyme inhibitors have been discovered through SBVS [17]. Several inhibitors of nematode chitinases were discovered by virtual screening [18]. In addition, through SBVS and experimental verification, a novel CYP1B1 inhibitor was discovered [19]. SBVS has opened up new avenues for enzyme inhibitor designing. However, to our knowledge, there has only been one report on Chitin deacetylase (CDA) inhibitor screening so far [20].

CDA belongs to the family 4 carbohydrate esterases, which catalyze the deacetylation of chitin to chitosan and participates in significant physiological and metabolic processes of fungi such as nutritional absorption, cell wall formation, spore formation, self-dissolution, and adhesion of mycelial larvae to the matrix [21, 22]. Deacetylation of chitin oligomers by CDA is used in preventing chitin-triggered host immunity [23, 24]. CDAs are widely distributed in nature but are absent in humans and plants [25]. Therefore, CDA has become an ideal target for fungi control, and several 3D crystal structures of CDA by X-ray diffraction were reported previously [26, 27]. The 3D structure of 3 kinds of fungi CDAs was deposited in the Protein Data Bank at the Research Collaboratory for Structural Bioinformatics, which provides essential information for the development of CDA-specific inhibitors.

In this study, the 3D structure of *An*CDA (pdb code: 2Y8U) was applied as the most reliable docking structure and used to screen the ChemDiv library through the SBVS method. The molecular mechanism of the compound J075-4187 was also investigated based on molecular dynamics simulation. Following drug-likeness, water solubility, drug interaction risk, and other indicators [28] , 25 compounds were selected for the study.

## Materials and Methods

### Strains, Vectors, and Chemicals

*Escherichia coli* DH5α was used as an intermediate host to construct plasmids. *Pichia pastoris* GS115 was used as a recipient strain for gene expression. Microorganisms used for the evaluation of the antifungal activity of compound J075-4187 of the ChemDiv compound library were: *Aspergillus nidulans* (ATCC 10074), *Fusarium graminearum* (ATCC 56091), *Aspergillus flavus* (ATCC 204304), *Botrytis cinerea* (ATCC48340), *Fusarium oxysporum* sp. *cucumebrium* Owen (ATCC42357), and *Saccharomyces cerevisiae* (ATCC 4226). Test fungal strains were cultured in Potato Dextrose agar. *E. coli* DH5α was grown at 37°C in an LB medium. *P. pastoris* GS115 was grown at 30°C overnight at 280 rpm in YPD medium. The pPICZ A plasmid was used to construct an expression vector. The medium for the *E. coli* cells containing the plasmid was supplemented with ampicillin (100 μg/ml). *Eco*RI, *Not*I, and a gel extraction kit were obtained from TaKaRa (Japan). 4-Nitroacetanilide was purchased from Beijing Bailingwei Technology Co., Ltd., China. An EasySelectTM Pichia Expression Kit was obtained from Invitrogen Co. (USA). The top-ranked 30 compounds derived from the ChemDiv database by structure-based pharmacophore virtual screening were purchased from ChemDiv, Inc. (USA). Unless otherwise noted, analytical-grade reagents and solvents were obtained from commercial sources.

### Structure-Based Virtual Screening Pipeline

The virtual filtering was carried out on the workstation (CPU: 40; Memory: 64; SSD: 512G) installed with the Ubuntu Kylin 15.10 operating system. The virtual filtering software FRED was used to virtual filter the structure of CDA, in which SDF format was selected for the running parameters, such as save_component_scores selection for true, and hitlist size, while the other parameters were also kept in SDF format.

The 3D structure of CDA (pdb code: 2Y8U) was retrieved from the Brookhaven Protein Data Bank. All water molecules and non-polar hydrogen atoms were eliminated, and the Gasteiger–Marsili method computed atomic charges. The structure was minimized by the steepest descent algorithm implemented in GROMACS 5.1. The MOE-site finder software was used to find the suitable pocket for structure-based virtual screening. Substrate-binding residues of *An*CDA were defined as compound-binding sites in which the docking grid was generated. Grid point spacing was set at 1Å, and 20 grid points were taken in each direction. The docked compounds in the Specs database were prepared with the LigPrep panel. All other docking parameters were assigned to their default values. ChemDiv (Version 2019) database was chosen as the compound library for virtual screening. Then, the prepared compounds were docked to the aforementioned docking grid. The “Clustering Molecules” protocol inserted in Pipeline Pilot 7.5 was employed to achieve the cluster analysis and and analyze the molecular docking-predicted binding mode of the compound in complex with *An*CDA. Discovery Studio v3.1 and PyMOL v2.0 were utilized to visualize the ligand-receptor complexes.

FTabeTo ensured the global conformation of small molecules in the virtual screening process, and plug-ins were used in the Openeye software. Compounds were scored by ChemGauss Score (CGS). The 3000 top-ranked compounds were analyzed based on experimental data integrated by Stardrop software and scored with the critical role of the central nervous system affinity. Ultimately, the top 23 hits with different scaffolds were purchased and tested for inhibition of chitin deacetylase activity.

### Synthesis, Expression, and Purification of Recombinant *An*CDA

The codon of *A. nidulans* chitin deacetylase gene (GenBank: XM_677557.1) was synthesized by GenScript Biotech Co. (China). *An*CDA-*Eco*RI-F: GGAA TTCA TGTTCGCAACCCTGGCCCTGGTGT. *An*CDA-*Not*I-R: A TAAGAA TGCGGCCGCTTAATGATGGTGATGATGATGATGATACCAGGCAATTT. The synthesized *An*CDA gene was used as a template, and the above primers were used for PCR amplification. The PCR amplification conditions were as follows: 94°C 5 min, 32 cycles (94°C 30 s, 55°C 30 s, 68°C 30 s). The PCR product was purified with a Gel Extraction Kit. The purified DNA and pPICZ A plasmids were digested with *Eco*RI and *Not*I, respectively. The recombinant gene pPICZ A -*An*CDA was constructed by ligating the plasmid with T4 ligase and *An*CDA was transferred into *E. coli* DH5α. The recombinant *E. coli* DH5α was spread on the low-salt LB medium containing Zecoin (100 μg/ml) and cultured overnight at 37°C. The plasmid was extracted by a plasmid extraction kit and sent for sequencing.

After gene sequencing, plasmids were transformed into *P. pastoris* GS115, cultured in YPD medium until OD600 was 1.3-1.5, then re-suspended with sterile 1-mol/l sorbitol after centrifugation. The extracted plasmid was linearized and transformed into the *P. pastoris* GS115 strain by electroporation. The transformed cells were plated on YPD plates containing 100 μg/ml bleomycin and cultured at 28°C for 2 days. Single clones were selected for PCR amplification to confirm target gene insertion in the yeast genome.

A single colony was grown in 10 ml of buffered glycerol-complex medium (BMGY, 1% yeast extract, 2%tryptone, 1.34% yeast nitrogen base (YNB), 1% glycerol, 4×10–5% biotin, and 100 mM potassium phosphate buffer, pH 6.0) and cultured at 28°C and 200 rpm until an OD600 of 2.0–6.0 was obtained. The culture was centrifuged at 2,000 g for 5 min, and the supernatant was discarded. Fifty milliliters of BMMY liquid medium was used to resuspend the cells and was then cultured at 28°C and 200 ×*g* for 7 days with 0.5% methanol added every 24 h. The broth was centrifuged at 12,000 g for 10 min at 4°C. According to the manufacturer's manual, the supernatant was collected and purified by His60 Ni Gravity Column (Takara, Japan).

### Inhibitory Activity Assays

The test compound J075-4187was dissolved in 1% (v/v) DMSO while *An*CDA and the substrate (GlcNAc)5 were both dissolved in Tris-HCl buffer (pH 8.0). The compounds and *An*CDA were preincubated in Tris-HCl buffer (37°C, 15 min). The reaction mixtures of 5 ml containing 1 μM purified *An*CDA, 2 mM substrate, and 10 μM CoCl2 in 25 mM Tris-HCl buffer (pH 8.0), were shaken and incubated at 37°C for 2 h. Finally, the reactions were quenched by adding acetonitrile to mixtures at a final concentration of 50% (v/v), and acetate release was measured using the K-ACETRM Kit (Ireland). One unit of enzymatic activity was defined as the amount of enzyme liberating 1 μM acetic acid per minute.

Inhibitory activity (%) = [(Δcontrol-Δsample)/Δcontrol] × 100

### Kinetic Study on CDA Inhibition by Compound J075-4187

The inhibition type of the inhibitors against *An*CDA activities was evaluated by the previously reported method [29]. Increasing constant concentrations of substrates (GlcNAc)5 were used in the absence or presence of compound J075-4187 at three different concentrations. The inhibition constant (Ki) was calculated using Dixon plots by changing the compound concentration at 40 μM, 60 μM, and 80 μM of (GlcNAc)5.

### Antifungal Assay

The fungal test strains were cultured in PDA slant tubes at 30°C for 3 days. Spores were collected with 1 ml sterile saline and diluted to 105 spores/ml. Sterilized solid growth media in petri dishes (9 cm diameter) were swabbed uniformly with 0.5 ml of the culture as with the above-mentioned media (105 spores/ml) for disk diffusion assay. Sterile 6 mm filter paper discs were placed on the plates. Then, 10 μl of the examined compound in 5% DMSO (0.25, 0.5, 1.0 mg/ml) was added immediately. Sterile paper discs added with 10 μl of 5% DMSO were used as the control. The plates were left for 30 min at room temperature to allow compound diffusion and then were incubated at 30°C for 36 h. Inhibition zone diameters were measured in triplicate in millimeters. Values are presented as means ± SD [30].

The microdilution method was used for the assessment of minimum inhibitory concentration (MIC). Briefly, the fungal spores were washed from the surfaces of agar plates with sterile 0.85% saline. The spore suspension was adjusted with sterile saline to a concentration of approximately 1.0 × 10^5^ in a final volume of 100 μl/well. MIC determinations were carried out by a serial dilution technique using 96-well microtiter plates. The examined compound was diluted in 5% DMSO (1–1,000 μg/ml), added to a PDB medium with inoculum, and incubated for 72 h at 30°C. The lowest concentrations without visible growth analyzed with the binocular microscope were defined as MICs. The fungicidal concentrations (MFCs) were determined by serial subcultivations of 2 μl of well content into microtiter plates containing 100 μl of broth/well and further incubated for 72 h at 28°C. The MFC was defined as the lowest concentration with no visible growth. The clinically used fungicides bifonazole and ketoconazole were used as positive control.

### Statistical Analysis

Experiments were performed in triplicate unless otherwise specified. All data are expressed as mean ± SD. Statistical significance was performed with SPSS 13.0 to analyze variance (ANOVA), followed by Dunnett's test.

## Results and Discussion

### Compounds Obtained by Structure-Based Virtual Screening

**Fig. 1:**
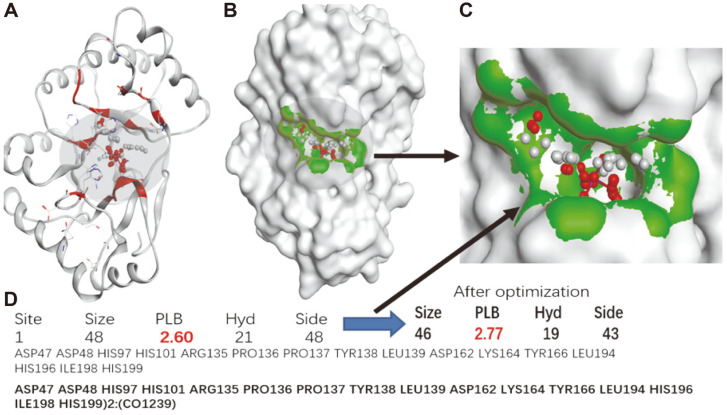
CDA (R26-H237) based on protonated treatment and 3D structures and small molecules after structural optimization. The 3D structure (**A**) of the CDA protein and the surface area (**B**), the shape and size of the smallmolecule binding pocket (**C**), the attributes of the small-molecule binding pocket (**D**).

After protonation and structural optimization of the crystal structure 2Y8U, a potential small molecule binding pocket located in the cobalt ion binding region was found on the surface of *An*CDA. The pocket contained 46 atoms, so the pocket theoretically tends to bind compounds with moderate molecular weight. The pocket also contained 19 atoms with strong hydrophobicity. The comprehensive score of the pocket as a drug binding was 2.77 (a value greater than 0 is acceptable). The pocket contained the following amino acids: Asp47, Asp48, His97 His101 Arg135 Pro136 Pro137 Tyr138 Leu139 Asp162 Lys164 Tyr166 Leu194 His196 Ile198 His199 (Fig. 1).

In addition, 3,000 small molecular compounds of CDA with the best interaction energy between receptor/protein and small molecules were obtained. The CGS distribution of their binding energy was from-14.08 to-10.89, and the molecular weight distribution was from 137 Da to 712 Da. The properties of the small molecular compounds were analyzed based on experimental data integrated by Stardrop software, such as water-solubility (logS), lipid-water distribution coefficient (logP), molecular weight, molecular flexibility, hydrogen bond properties, surface accessibility area (TPSA), CYP2C9 enzyme degradation level, hERG inhibition rate index, oral utilization (HIA), drug interaction risk (2D6), and so on. Then, each small molecule was rated using the screening criteria for oral, non-central nervous system drugs and the affinity level. For the common substructure cluster analysis of these 3,000 small molecules, where the similarity was set to 0.5, 200 categories were calculated, and 473 compounds were selected based on structural diversity, CDA affinity, and comprehensive scoring values.

Through the common skeleton (common substructure) cluster analysis of these 473 compounds, the similarity was set to 0.7. A total of 43 categories were calculated, of which 61 compounds did not belong to any skeleton, and their compound structure space is shown in Fig. S1. Based on the comprehensive analysis of the structural diversity, CDA affinity, and drug score of the compounds, we selected 150 compounds with the best properties (the molecules with the best scores in each group) and the diversity skeletons (selected molecules in multiple groups). These compounds were distributed into 25 groups with different skeletons; they have high-affinity CDA, good water solubility, and they are also fat-soluble (logP) (Figs. S2 and S3). Then, the top 23 compounds with the best scores in each common substructure cluster were selected (Table 1).

### *An*CDA Inhibitory Activity

**Fig. 2:**
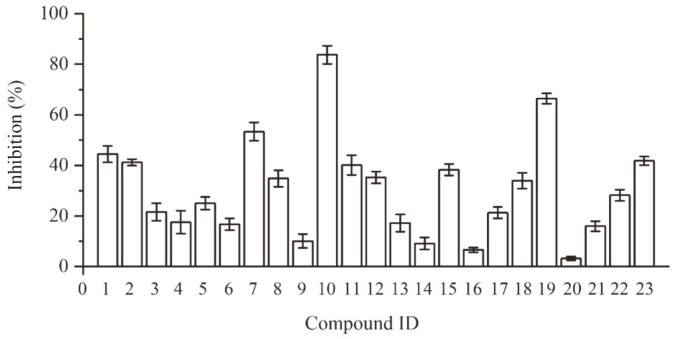
Effect of compounds on chitin deacetylase activity at 0.1 μM.

The selected 23 candidates were initially evaluated for their inhibitory ratios against *An*CDA at 100 μM. The results showed that all the candidates showed inhibition against *An*CDA, and compound J075-4187 showed the highest inhibitory activity of 83.77% against *An*CDA (Fig. 2). In recent years, significant progress has been made in identifying inhibitors targeting chitin–related enzymes. However, developing specific antifungal agents is tricky because chitinases are widely distributed [31].

Several inhibitors of carbohydrate esterase family 4 (CE-4) enzymes have been reported previously, including *Bacillus cereus* peptidoglycan *N*-acetylglucosamine deacetylase Bc1974 [32, 33], and peptidoglycan deacetylase (PgdA) from *Streptococcus pneumoniae* [34].

The research on the CDA inhibitors is limited. AcOH formed during the deacetylation process acts as a competitive inhibitor of CDA. For the CDA from *Mucor rouxii* ATCC 24905, 250 mM AcOH decreased the enzyme activity to 10% of the initial value. However, AcOH showed less significant influence on CDA from *Colletotrichum lindemuthianum* ATCC 56676 [35].

### Kinetic Study on Chitin Deacetylase Inhibition by Compound J075-4187

**Fig. 3:**
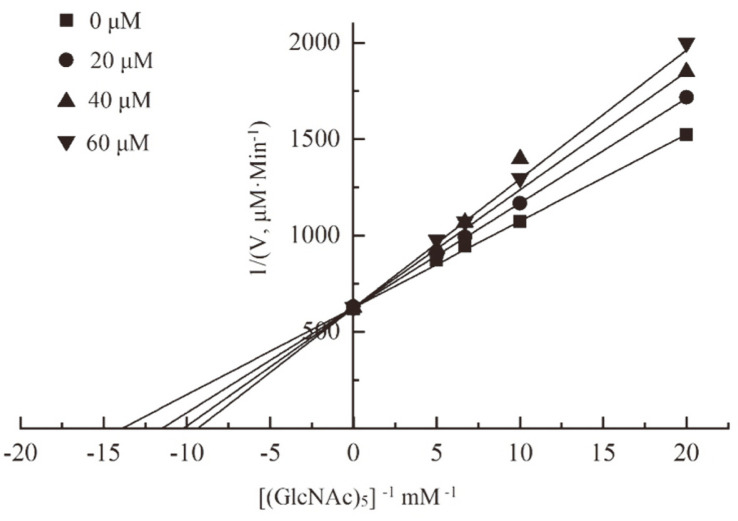
Kinetic assay on *An*CDA inhibition by compound J075-4187.

**Fig. 4:**
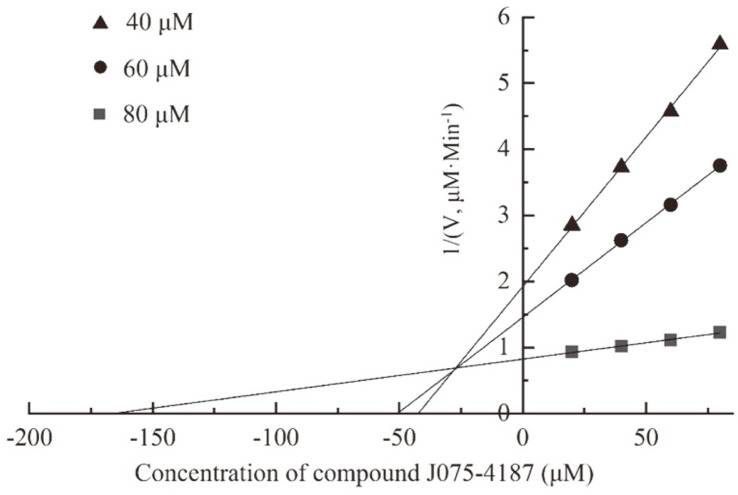
Kinetics of compound J075-4187 against *An*CDA determined by Dixon plot analysis.

A kinetic analysis was performed to explore the mechanism of the interaction of J075-4187 with the enzyme. As shown in Fig. 3, in the presence of compound J075-4187, an increase in Km and a constant in Vmax were observed, indicating that the compound J075-4187 was a competitive inhibitor of *An*CDA. It indicated that compound J075-4187 could bind to *An*CDA with high affinity and prevent substrate binding to the active site of *An*CDA. The Dixon plot obtained is typical for competitive inhibition. The inhibition constant (Ki) was calculated using Dixon plots by changing the compound concentration at 40 μM, 60 μM, and 80 μM of (GlcNAc)5. The estimated Ki value of compound J075-4187 against *An*CDA was 31.58 μM (Fig. 4). The Km of *An*CDA for (GlcNAc)5 was 72 μM and 1.4 s−1. The significant difference in values of Km and Ki suggests that the enzyme may have a greater affinity for the inhibitor than for the substrate. The inhibitor will bind to the enzyme more quickly than the substrate in the reaction mixture. CDA does not exist in humans or plants, so inhibitors targeting CDA are promising green fungicides. CDA could also be used as a potential pest control target [36].

Finding inhibitors against chitin deacetylase will also help to clarify their structural features and mechanism. Additionally, successful inhibitors would have biological advantages.

### Molecular Docking Simulation Revealed the Binding Mode of Compound J075-4187

**Fig. 5:**
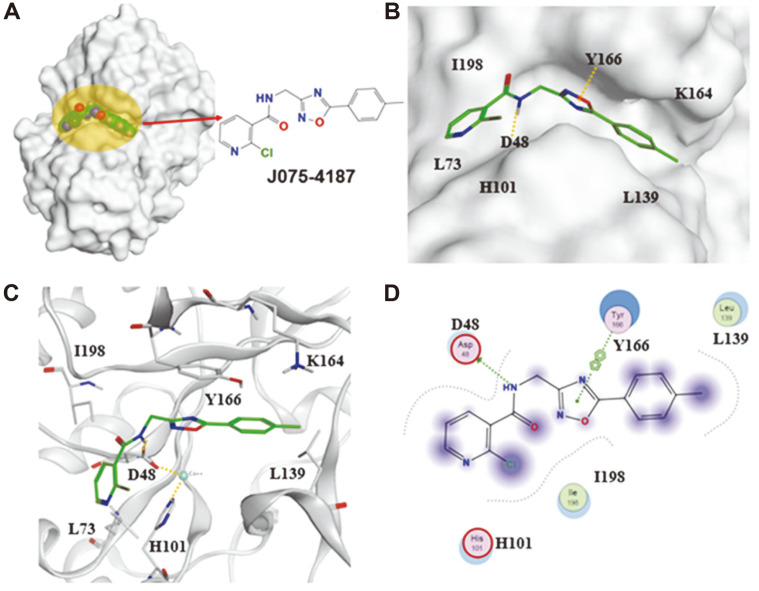
Docking pose of compound J075-4187 bound to the chitooligosaccharides binding site in *An*CDA. Amino acid D48 forms a hydrogen bond interaction with the compound, and combines cobalt ion with H101. Amino acids L73, L139, K164, Y166, I198 and other adjacent compounds may have van der Waals forces. (**A**) The molecular surface area diagram of the CDA-compound complex. The orange area is the small-molecule binding area, and the compound is displayed with a 2D structure and a green spherical structure. (**B**) The binding mode diagram of the compound in the CDA-binding pocket, the orange dashed line indicates the interaction between atoms. (**C**) Cartoon diagram of the interaction between the compound and the CDA target. (**D**) The 2D diagram of the interaction between the compound and the target.

To predict the inhibitory mechanism of the J075-4187 compound to chitin deacetylase, its molecular docking-predicted binding mode to the *An*CDA crystal structure was analyzed. Molecular dynamics simulations were performed on the small molecule compound J075-4187. As shown in Fig. 5, compound J075-4187 could bind well to the chitooligosaccharide sites, including the active sites (H101 and D48). The active sites of *An*CDA include the His-His-Asp metal-binding triad (H97, H101, and D48), a catalytic acid (His196, aiding sugar departure), and a catalytic base (Asp47).

Moreover, D48 and H101 participate in stabilizing the complex through the metal cobalt ion. Amino acids L73, L139, K164, Y166, and I198 may have van der Waals forces with compound J075-4187. These results were consistent with the competitive property. They also provide evidence for the CDA binding motif and a basis for further engineering compound J075-4187 to compounds with improved CDA binding for future applications. Amino acid D48 forms a hydrogen bond interaction with the compound and combines cobalt ion with H101.

### Antifungal Activity of Compound-J075-4187 to Representative Food Spoilage and Plant Pathogenic Fungi

**Fig. 6:**
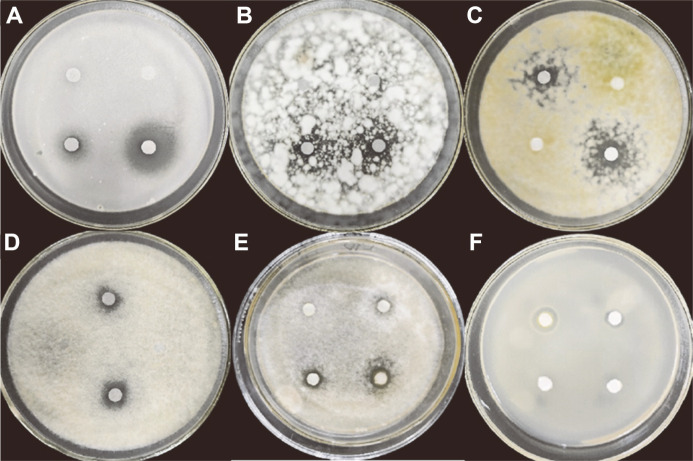
Antifungal activity of compound-J075-4187 to representative food spolige and plant pathogenic fungi. (**A**) *A. nidulans*; (**B**) F. graminearum; (**C**) A. flavus; (**D**) B. cinerea Persoon; (**E**) Fusariumo xysporum f. sp. cucumebrium Owen; (**F**) *S. cerevisiae*.

The antimicrobial activity of compound J075-4187 to representative food spoilage and plant pathogenic fungi was determined. As shown in Fig. 6, the compound J075-4187 produced an apparent inhibition zone for all tested samples, including plant pathogenic fungi and food spoilage fungi. However, the diameters of inhibition zones are different from species to species. The largest diameter inhibition zone was observed against *A. nidulans*, while the minimum diameter was observed for *S. cerevisiae* (Table 2). The compound J075-4187 exhibited activity for all six fungal strains. The best activity was against *A. nidulans* with MIC at 60 μg/ml, and MBC at 100 μg/ml, respectively. While the lowest activity was shown against *S. cerevisiae* with MIC at 260 μg/ml, and MBC at 520 μg/ml, respectively. Further research of the mechanism for the compound J075-4187 inhibiting the growth of *A. nidulans* is ongoing.

Plant pathogenic fungi cause significant economic losses to crops worldwide, with estimated loss of $40 billion [37]. Synthetic fungicides are used to control pathogenic fungi in crops [38]. However, their utilization has several drawbacks due to fungal resistance and adverse effects on human and animal health and the environment in general [39]. These concerns have been the main driving force of research to identify eco-friendly methods for managing fungal diseases. CDA participates in important physiological and metabolic processes of fungi. The compound J075-4187 inhibits the activity of CDA and leads to the inhibition of fungi, and could be used to develop new environmental fungicides to control plant diseases [40].

The compound J075-4187 was screened from the commercial Chemdiv compound library based on SBVS and biological evaluation. Molecular docking calculations predicted that compound J075-4187 binds to the chitooligosaccharide sites, including active sites (H101, D48). The compound J075-4187 inhibited food spoilage fungi and plant pathogenic fungi, with minimum inhibitory concentration (MIC) at 260 μg/ml and minimum fungicidal concentration (MFC) at 520 μg/ml, and could be used as a candidate for the development of antifungal agents for fungi control. To the best of our knowledge, few CDA inhibitors have been reported, and no antifungal drugs have been developed based on CDA inhibitors. The compound J075-4187 could serve as a lead compound for the development of new novel inhibitors, which could be developed into antifungal drugs.

## Supplemental Materials

Supplementary data for this paper are available on-line only at http://jmb.or.kr.

## Figures and Tables

**Table 1:** Representative molecular properties and key parameters identified in docking-based VS of the 23 purchased compounds from ChemDiv database.

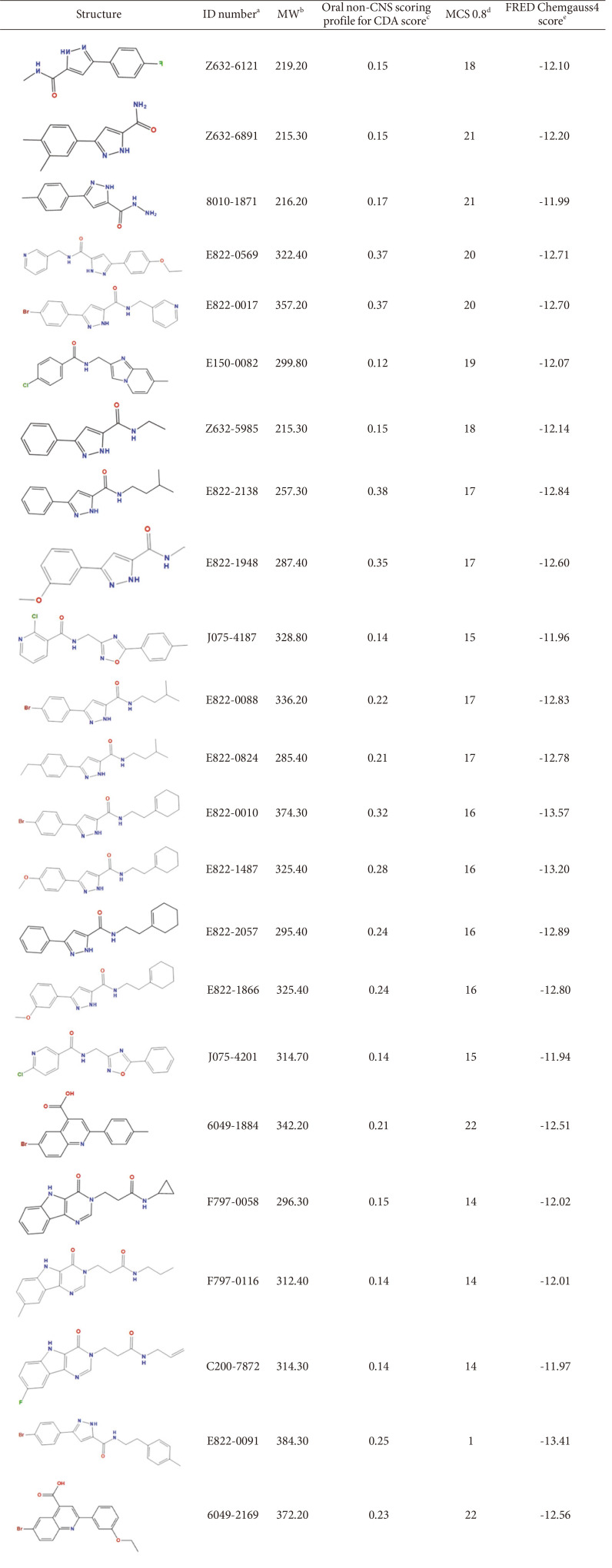					

Data are expressed as geometric mean values of six runs ± the standard error of the mean (SEM).

The compound number labeled in the ChemDiv database. According to the purity statements, the purity of all compounds purchased from the ChemDiv database is higher than 95%.

^b^Molecular weight.

^c^Through the scoring function, affinity, water solubility (logS), lipid-water distribution coefficient (logP), molecular weight, molecular flexibility, hydrogen bond properties, surface accessibility area, CYP2C9 enzyme degradation level, hERG inhibition rate, oral utilization rate, drug interaction A comprehensive evaluation of the risk of action (2D6) and other attributes. The value range is 0-1. The higher the score, the better the affinity and druggability of the compound.

^d^Small molecule compounds are clustered by a skeleton similarity of 0.8, and molecules with the same skeleton have the same MCS value.

^e^The binding energy of the compound and CDA. The lower the value, the stronger the binding ability.

**Table 2:** Antifungal activity of compound J075-4187.

Test fungi	Inhibition zone (mM)	MIC (μg•ml^-1^)	MFC (μg•ml^-1^)
*Aspergillus nidulans*	17.16 ± 1.26	60 ± 0.010	100 ± 0.020
*Fusarium graminearum*	9.88 ± 2.08	120 ± 0.006	210 ± 0.008
*Aspergillus flavus*	13.06 ± 1.70	100 ± 0.020	160 ± 0.012
*Botrytis cinerea* Persoon	8.92 ± 2.02	130 ± 0.016	250 ± 0.008
*Fusariumo xysporum.* Sch1f. sp. *cucumebrium* Owen	9.24 ± 1.86	120 ± 0.022	230 ± 0.022
*Saccharomyces cerevisiae*	7.38 ± 1.28	260 ± 0.008	520 ± 0.016
