# Supplementary material for: Discovery of Chitin Deacetylase Inhibitors through Structure-Based Virtual Screening and Biological Assays
Source: J Microbiol Biotechnol. 2022 Feb 5;32(4):504–13. doi: 10.4014/jmb.2201.01009 (PMC9628821; doi:10.4014/jmb.2201.01009)
Supplement: Supplementary file 1 [file jmb-32-4-504-supple.pdf]

J. Microbiol. Biotechnol.
https://doi.org/10.4014/jmb.2201.01009

Supplementary Figures

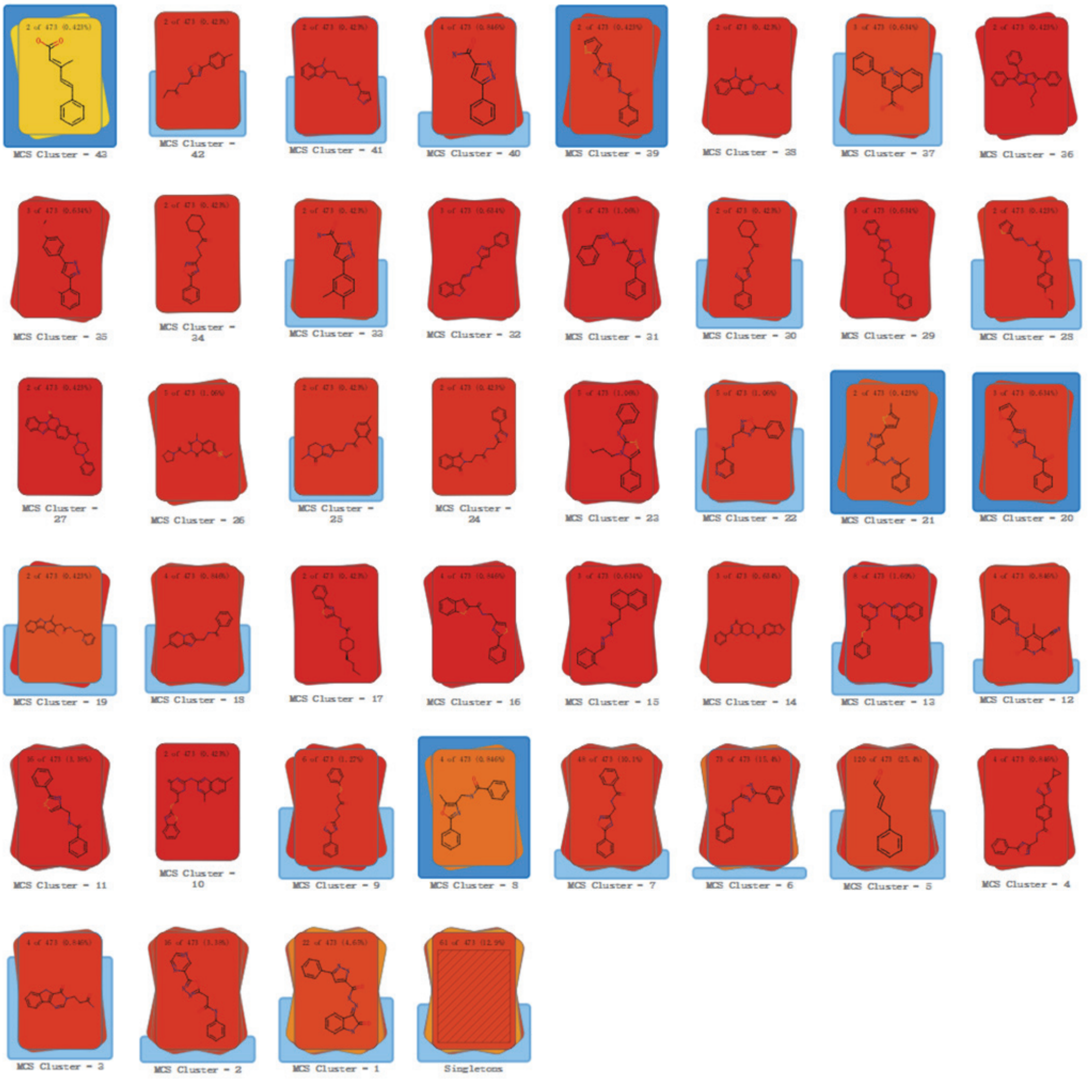

Supplementary Figure 1. Spatial structure information of 43 types of compounds. The scoring value of druggability decreases gradually from yellow to red, and the blue background is the selected group.

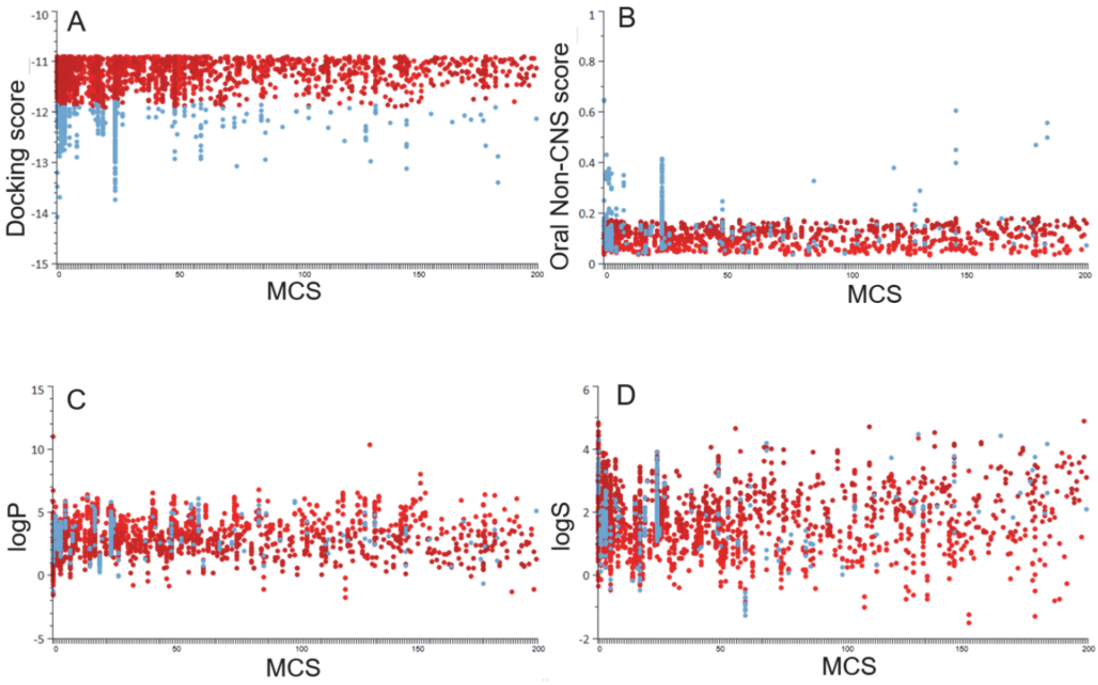

Supplementary Figure 2. Correlation analysis chart of comprehensive score and different properties of 3000 compounds in 200 categories. The correlation analysis chart of small molecule compounds and their affinity CDA score (a); the correlation analysis chart of small molecule compounds and their comprehensive score (b); the correlation analysis chart of small molecule compounds and their fat solubility (c); the correlation analysis chart of small molecule compounds and their water solubility (d). The score of small molecule compounds decreased from black to red. Blue is the final selection of 473 small molecule compounds.

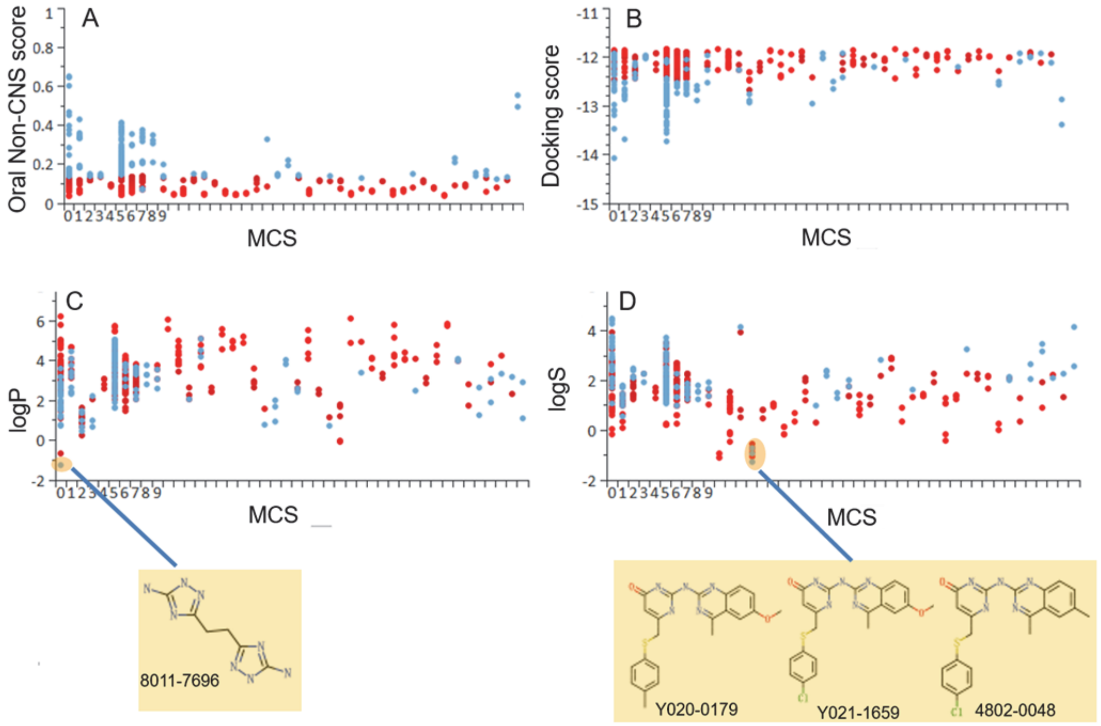

Supplementary Figure 3. Comprehensive score values of a total of 473 compounds in 43 classes and correlation analysis plot of their different attributes.The grouping situation of small molecule compounds and their comprehensive score value (a); the grouping situation of small molecule compounds and correlation analysis plot of their affinity CDA score value (b); the grouping situation of small molecule compounds and correlation analysis plot of their lipid solubility (c); the grouping situation of small molecule compounds and correlation analysis plot of their water solubility (d).Where the druggability score value of small molecule compounds is decreasing in a gradient from black to red in color.Blue for the final selected 150 small molecule compounds.
